# Always Consider a Repeat Kidney Biopsy: Acute Interstitial Nephritis Soon After Membranous Nephropathy

**DOI:** 10.1155/crin/2277958

**Published:** 2026-01-31

**Authors:** Jarrad Hopkins, James Nolan, Maleeka Ladhani, Chiang Lee

**Affiliations:** ^1^ Faculty of Health and Medical Sciences, The University of Adelaide, Adelaide, South Australia, Australia, adelaide.edu.au; ^2^ Division of Medicine, Renal Unit, Lyell McEwin Hospital, Elizabeth Vale, South Australia, Australia, sahealth.sa.gov.au; ^3^ Department of Anatomical Pathology, SA Pathology, Adelaide, Australia, sapathology.sa.gov.au

**Keywords:** acute interstitial nephritis, membranous nephropathy, repeat kidney biopsy

## Abstract

**Introduction:**

The heterogeneity of membranous nephropathy is well described in the literature, and its clinical course and response to treatment vary. Similarly, acute interstitial nephritis (AIN) can present in unexpected and unusual ways and should always be considered within the differential diagnosis of worsening renal function. This case study describes the cross‐section of these two disease entities.

**Clinical Case:**

A 68‐year‐old male with a history of hypertension, chronic obstructive pulmonary disease (COPD), hyperlipidaemia, gout and treated prostate cancer presented with bilateral lower limb swelling and progressive renal dysfunction. Initial laboratory findings demonstrated nephrotic syndrome with impaired renal function. A positive phospholipase A2 receptor (PLA2R) antibody confirmed primary membranous nephropathy. Renal biopsy showed typical findings of membranous nephropathy. Treatment with prednisolone and cyclophosphamide improved renal function initially. However, as prednisolone was tapered, creatinine levels rapidly worsened, leading to a second biopsy. The second biopsy demonstrated AIN superimposed on chronic membranous nephropathy. A drug‐induced aetiology was suspected, with pantoprazole, trimethoprim–sulfamethoxazole and frusemide identified as the most likely contributors. Following withdrawal of these agents and reinitiation of high‐dose corticosteroids, the patient’s renal function improved markedly, obviating the need for dialysis.

**Conclusion:**

This case highlights the importance of considering AIN and the need for timely repeat renal biopsy in cases of deteriorating renal function.

**Summary**

• This case highlights the complexity of diagnosing dual pathology and the importance of repeat kidney biopsy. The presence of primary **PLA2R**‐positive membranous nephropathy as the initial diagnosis, followed by acute interstitial nephritis (AIN) likely triggered by medications, emphasises the importance of Hickam’s dictum: A patient can have more than one disease process.

## 1. Introduction

Membranous nephropathy is the most common cause of nondiabetic nephrotic syndrome in adults [1]. One of the challenges of this disease is the heterogeneity of its presentation and clinical course. The phospholipase A2 receptor (PLA2R), which is abundantly expressed on podocytes, is targeted by circulating autoantibodies in up to 80% of cases of primary membranous nephropathy. The addition of PLA2R antibody titres is a valuable tool in measuring and monitoring disease activity [2]. The disappearance of PLA2R antibodies in circulation defines immunological remission and is a strong independent predictor of subsequent clinical remission [3].

Acute interstitial nephritis (AIN) is identified in approximately 15%–27% of kidney biopsies performed for evaluation of acute kidney injury [4]. Its aetiologies are diverse and include drug‐induced, infection‐related, idiopathic and systemic disease–associated causes [5]. A definitive diagnosis via kidney biopsy is necessary because noninvasive tests and imaging lack sensitivity and specificity [6]. Biopsy confirmation also guides management, particularly the timely withdrawal of potential offending agents.

Here, we describe a unique case of drug‐induced AIN occurring in the context of treatment for membranous nephropathy.

## 2. Case Report

A 68‐year‐old Caucasian male presented to his general practitioner with bilateral lower limb swelling over 4 weeks. His past medical history includes hypertension, hyperlipidaemia, chronic obstructive pulmonary disease (COPD), gout, gastro‐oesophageal reflux disease (GORD) and recent prostate cancer treated with complete prostatectomy 5 months ago. His relevant medications included allopurinol, bisoprolol, citalopram, fenofibrate, lercanidipine and esomeprazole. He had a systolic blood pressure of 190 mmHg and bilateral lower limb oedema to upper thighs. There were no other clinical manifestations to suggest disease aetiology. On presentation, he had significant renal impairment with serum creatinine 302 µmol/L (last result 150 µmol/L 1 year prior), urea 11.1 mmol/L, potassium 4.1 mmol/L, albumin 18 g/L, C‐reactive protein 1.9 mg/L, haemoglobin 129 g/L and total cholesterol was 4.8 mmol/L. Urine assessment showed urine protein to creatinine ratio (PCR) 812 mg/mmol and no microhaematuria. Furthermore, 24‐h urinary protein was 8.3 g in 24 h, supportive of likely nephrotic syndrome.

Results (Table 1) showed that he had no detectable antinuclear antibody (ANA), double‐stranded DNA (dsDNA) antibody and extractable nuclear antigen (ENA) antibody, and rheumatoid factor was negative. Hepatitis C virus, hepatitis B virus surface antigen and HIV serology were noncontributory. Furthermore, his prostate‐specific antigen (PSA) was undetectable < 0.01. Serum C3 and C4 were normal. There was no detectable monoclonal paraprotein, and the serum *κ* and *λ* free light chains (FLCs) were proportionally elevated, *κ* 107.12 mg/L (3.3–19.4 mg/L) and *λ* 100.05 mg/L (5.7–26.3 mg/L), respectively, with *κ*/*λ* FLC ratio at 1.07 (0.26–1.65 *κ*/*λ*) consistent with renal impairment. Serum PLA2R antibody was strongly positive at 359.9 IU/mL (< 13.9 IU/mL), suggestive of primary membranous nephropathy.

**Table 1:** Summary of results.

Test	Value
Creatinine	320 µmol/L
Urea	11.1 mmol/L
Potassium	4.1 mmol/L
Albumin	18 g/L
Haemoglobin	129 g/L
Total cholesterol	4.8 mmol/L
Urine protein creatinine ratio (PCR)	812 mg/mmol
24‐h urinary protein	8.3 grams
Antinuclear antibody (ANA)	Negative
Double‐stranded DNA (dsDNA) antibody	Negative
Extractable nuclear antigen (ENA)	Negative
Hepatitis screen and HIV	Negative
Prostate‐specific antigen (PSA)	< 0.01
C3 and C4	Normal
Phospholipase A2 receptor antibody	359.9 IU/mL (RR < 13.9 IU/mL)
Paraprotein	0 g/L
*κ*/*λ* FLC ratio	1.07

**Figure 1:**
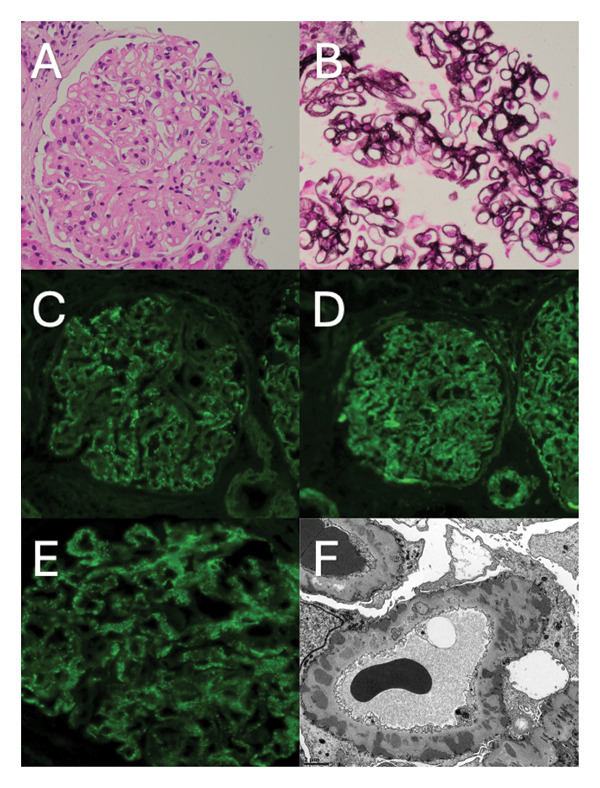
Glomeruli showed marked capillary wall thickening (A), and in the silver stains, there is focal membrane splitting and double contouring and features consistent with vacuolar alteration to the basal membrane and occasional spikes are seen (B). Immunofluorescent labelling for IgG (C), C3 (D) and IgA (E) showed granular capillary wall deposition. Electron microscopy showed all four stages of subepithelial electron‐dense deposits within the glomerular basement membranes (F).

Renal biopsy (Figure 1) showed glomeruli with uniform, marked capillary wall thickening with focal membrane splitting and double contouring with occasional spikes. There are areas of chronic scarring varying from 20% to 70% in different regions of the cortex with tubular atrophy. By immunofluorescence, there was granular staining of capillaries and mesangium for IgA, IgG and both 1+ and C3 2+. Electron microscopy (EM) was consistent with membranous nephropathy, with all four stages of subepithelial electron‐dense deposits represented.

The patient was diagnosed with primary PLA2R‐positive membranous nephropathy, and he was commenced on immunosuppression consisting of prednisolone 50 mg with plans to wean gradually over several weeks and oral cyclophosphamide 100 mg daily. Oral cyclophosphamide is preferred in our centre when there is heavy proteinuria, such as in this case. He was also commenced on irbesartan 75 mg daily, prazosin 0.5 mg BD, trimethoprim/sulfamethoxazole 160 mg/800 mg 0.5 tablets three times a week (pneumocystis jirovecii prophylaxis), frusemide 40 mg daily and pantoprazole 40 mg daily (esomeprazole was stopped). He had never previously been exposed to these agents.

Over the next 12 weeks (Table A1), his symptoms resolved, renal function improved (creatinine 190–200 µmol/L) and proteinuria (urine PCR 70 mg/mmol) improved. However, around Week 13, as the prednisolone was reduced to 15 mg daily, his renal function deteriorated from 238 µmol/L to 422 µmol/L prompting hospital admission for evaluation. Clinically, his blood pressure was 160/90 mmHg, and he had mild fluid overload. Investigations revealed rapidly progressive renal failure with worsening serum creatinine from 422 µmol/L to 651 µmol/L over a span of 3 days, persisting hypoalbuminemia with albumin 23 g/L with worsening proteinuria (urine PCR 322 mg/mmol). There was no evidence of rash, eosinophilia or sterile pyuria. He had an ultrasound renal tract that did not show any evidence of obstruction or renal vein thrombosis. PLA2R antibody was negative as was antibodies to myeloperoxidase (MPO), proteinase 3 (PR3) and glomerular basement membrane (GBM). The authors felt it was important to investigate with a repeat kidney biopsy.

**Figure 2:**
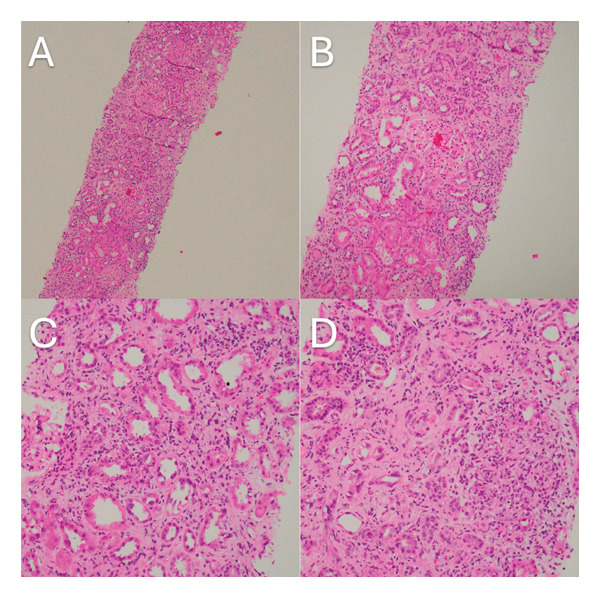
Widespread inflammatory infiltrate and oedema with infiltrate (A, B) including lymphocytes and eosinophils, the latter are focal. The lymphocytes extend into tubules causing tubulitis, (C) and a focal granuloma is seen (D). There is significant background scarring of the cortical parenchyma.

The second renal biopsy (Figure 2) showed widespread inflammatory infiltrate and oedema. The infiltrate includes lymphocytes and eosinophils, which are focal. They extend into the tubules, and focally, a granuloma is identified. The previous identified glomerular pathology of membranous nephropathy is also seen. There is 60% scarring in the cortex, and the immunofluorescence findings were similar but weaker; there was granular staining of capillaries and mesangium for IgA, IgG and C3 1+. The renal biopsy was consistent with AIN on a background of scarring and membranous nephropathy.

**Figure 3:**
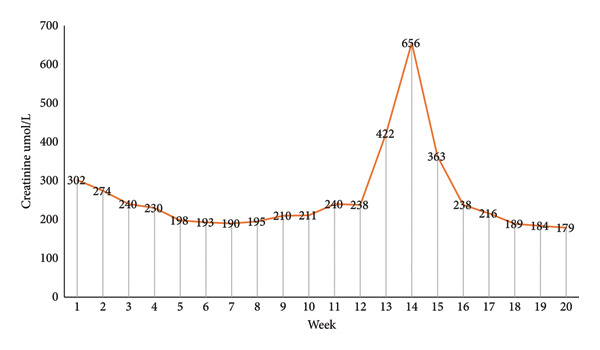
Creatinine over time.

Medications responsible for AIN included pantoprazole, trimethoprim/sulfamethoxazole and frusemide, and these were all ceased and replaced with famotidine (as gastroprotection) and nebulised pentamidine (pneumocystis jirovecii prophylaxis). His dose of prednisolone was increased to 50 mg daily, and fortunately, the creatinine peaked at 656 µmol/L, and he did not require haemodialysis. One month later, his creatinine improved to 170 µmol/L (Figure 3), and 6 months later his creatinine remains stable 170 µmol/L. His 24‐h urinary protein is 900 mg, and his PLA2R antibody is negative, he is in clinical remission, and he remains closely followed up by his nephrologist.

## 3. Discussion

This case study describes a case of drug‐induced AIN following the commencement of treatment for primary membranous nephropathy. There is a paucity of research, and we believe this to be a unique case. It also highlights the importance of a repeat kidney biopsy when there is diagnostic uncertainty. A repeat kidney biopsy is well described for patients with lupus nephritis as histological remission has been proven to have a more favourable outcome. Within the literature on lupus nephritis [7, 8], a repeat biopsy is done either ‘per‐protocol’ to evaluate the initial phase of induction immunosuppression or when there is a suspicion for a flare of disease; the latter is similar to our case. Our case highlights the benefits of a repeat renal biopsy during an atypical course of membranous nephropathy. There is one other case report in the literature where a patient developed postinfectious glomerulonephritis following community‐acquired pneumonia in the setting of immunosuppression from membranous nephropathy [9].

Interestingly, despite a negative PLA2R antibody, our patient still has histological features of ongoing membranous nephropathy with classical glomerular change and ultrastructural deposits seen on the second biopsy. There is a paucity of research on repeat biopsies in membranous nephropathy, so it is unclear whether the persistence of membranous immune deposits 3 months posttreatment initiation is expected. This would be a valuable area of further research, in particular in those who achieve PLA2R immunological remission such as our patient.

In the presence of membranous nephropathy with sudden worsening of renal function, it is important to exclude other pathologies such as malignant hypertension, hypovolaemia from diuresis or renal vein thrombosis [10]. These were systematically excluded in the patient by a thorough clinical examination and urgent renal ultrasound with a Doppler. With respect to dual pathologies, there are a number of case reports describing combined crescent glomerulopathy with positive ANCA or anti‐GBM antibodies [11], which were fortunately negative in our case. Similarly, one case report identifies interstitial nephritis and membranous disease coexistent in the setting of IgG4‐related disease [12], which was not seen in our case.

Drug‐induced AIN is the most common type, accounting for up to 75% of cases, with the most common culprits: antibiotics, nonsteroidal anti‐inflammatory drugs and proton‐pump inhibitors (PPIs) [13]. It is unclear which offending medication caused the acute interstitial process in our case, and in particular, rechallenging or other in vitro tests such as drug‐induced lymphocyte stimulation tests were not preformed. PPI‐associated AIN has become one of the leading causes of drug‐induced AIN in many settings owing to their wide and prolonged use [14]. Epidemiological data suggest a crude incidence rate of approximately 12 per 100,000 person‐years in PPI users [15]. Brewster et al. showed that PPI‐associated AIN, presentation with kidney involvement, occurred on average 10 weeks (range 1 week–9 months) after commencing PPI therapy [16]. Notably, our patient changed from esomeprazole to pantoprazole at a similar time prior to presentation.

## Ethics Statement

The authors have nothing to report.

## Consent

Written informed consent was obtained from the patient for publication of the case report including images. A copy of the written consent is available for review by the editor of this journal.

## Disclosure

All authors have read and approved the manuscript.

## Conflicts of Interest

The authors declare no conflicts of interest.

## Author Contributions

Jarrad Hopkins performed the background research and was the primary writer of the manuscript. Chiang Lee and Maleeka Ladhani were involved in the clinical care of the patient. James Nolan provided the images and interpretation of the kidney biopsies. Chiang Lee and Maleeka Ladhani provided substantial revisions to the manuscript.

## Funding

No funding was received for this manuscript.

## Data Availability

Data sharing is not applicable.

## Appendix 1

**Table A1:** Timeline of events.

Timeline	Event
January 2024	Prostate cancer surgery Baseline Cr 150, normal albumin and no proteinuria measured. No oedema or difficult to control hypertension. Regular medications: allopurinol, bisoprolol, citalopram, fenofibrate, lercanidipine and esomeprazole

May 2024	Symptoms developed—peripheral oedema and GP noted hypertension

June 2024	Presented to renal unit. Worsening renal function Cr 300 with 8 g proteinuria in 24‐h collection Biopsy performed—membranous nephropathy Commenced medications: prednisolone 50 mg (weaned) daily, cyclophosphamide 100 mg daily, irbesartan 75 mg daily, prazosin 0.5 mg BD, trimethoprim/sulfamethoxazole 160 mg/800 mg 0.5 tablet three times a week, frusemide 40 mg daily and pantoprazole 40 mg daily

September 2024	Asymptomatic. Currently on 15 mg prednisolone. Routine blood monitoring showed Cr rising Admitted for biopsy—showing acute interstitial nephritis Steroids increased to 50 mg Pantoprazole, trimethoprim/sulfamethoxazole and frusemide were all ceased and replaced with famotidine and nebulised pentamidine

October 2024	Creatinine returned to baseline 160–190

March 2025	Creatinine stable, 900 mg proteinuria and PLA2R negative. Clinical and laboratory remission
